# Spatial patterns of benthic biofilm diversity among streams draining proglacial floodplains

**DOI:** 10.3389/fmicb.2022.948165

**Published:** 2022-08-08

**Authors:** Jade Brandani, Hannes Peter, Susheel Bhanu Busi, Tyler J. Kohler, Stilianos Fodelianakis, Leila Ezzat, Grégoire Michoud, Massimo Bourquin, Paraskevi Pramateftaki, Matteo Roncoroni, Stuart N. Lane, Tom J. Battin

**Affiliations:** ^1^River Ecosystems Laboratory, Alpine and Polar Environmental Research Center, Ecole Polytechnique Fédérale de Lausanne (EPFL), Lausanne, Switzerland; ^2^Systems Ecology Group, Luxembourg Center for Systems Biomedicine, University of Luxembourg, Esch-sur-Alzette, Luxembourg; ^3^Institute of Earth Surface Dynamics (IDYST), University of Lausanne, Lausanne, Switzerland

**Keywords:** proglacial floodplains, benthic biofilms, microbial diversity, 16S and 18S rRNA amplicons, climate change

## Abstract

Glacier shrinkage opens new proglacial terrain with pronounced environmental gradients along longitudinal and lateral chronosequences. Despite the environmental harshness of the streams that drain glacier forelands, their benthic biofilms can harbor astonishing biodiversity spanning all domains of life. Here, we studied the spatial dynamics of prokaryotic and eukaryotic photoautotroph diversity within braided glacier-fed streams and tributaries draining lateral terraces predominantly fed by groundwater and snowmelt across three proglacial floodplains in the Swiss Alps. Along the lateral chronosequence, we found that benthic biofilms in tributaries develop higher biomass than those in glacier-fed streams, and that their respective diversity and community composition differed markedly. We also found spatial turnover of bacterial communities in the glacier-fed streams along the longitudinal chronosequence. These patterns along the two chronosequences seem unexpected given the close spatial proximity and connectivity of the various streams, suggesting environmental filtering as an underlying mechanism. Furthermore, our results suggest that photoautotrophic communities shape bacterial communities across the various streams, which is understandable given that algae are the major source of organic matter in proglacial streams. Overall, our findings shed new light on benthic biofilms in proglacial streams now changing at rapid pace owing to climate-induced glacier shrinkage.

## Introduction

Glacial forelands figure among the geomorphologically most dynamic landscapes on Earth, and their streams impact the biogeochemistry and sediment dynamics of downstream ecosystems (Anderson, 2007; Heckmann et al., 2016). Climate-induced glacier shrinkage is currently accelerating the formation of glacial forelands (Heckmann et al., 2016), promoting the development of biogeochemical and ecological gradients along longitudinal and lateral chronosequences (Bormann and Sidle, 1990; Ficetola, 2021). Floodplains are conspicuous features of many glacial forelands, and feature both braided channel networks directly fed by glacier meltwaters, as well as tributary channels that drain lateral terraces, which are typically fed by groundwater and snowmelt (Malard et al., 2000; Brown et al., 2007). This co-occurrence of diverse stream types, differing in channel morphodynamics, hydrological regimes, water sources and physicochemical characteristics, is facilitated by the geomorphological chronosequences. Collectively, the variety of stream types at relatively small special scales (i.e., few kilometers) results in remarkable environmental heterogeneity that promotes aquatic biodiversity at the level of the floodplain (Milner and Petts, 1994; Milner et al., 2010, 2017).

How habitat heterogeneity along chronosequences affects biodiversity has been relatively well studied for aquatic invertebrates (Milner and Petts, 1994; Milner et al., 2010; Cauvy-Fraunié and Dangles, 2019; Muhlfeld et al., 2020); however, less so for microorganisms. The bulk of our current understanding on the microbial life in proglacial streams remains limited to the main channels directly fed by glacier meltwaters (e.g., Battin et al., 2004; Wilhelm et al., 2013; Ren et al., 2017; Roncoroni et al., 2019; Busi et al., 2021). Some of these earlier studies have presented tentative evidence toward longitudinal shifts in community structure and function of benthic biofilms in glacier-fed streams (Battin et al., 2004; Ren et al., 2017). A noticeable exception to this linear perception is the work by Freimann et al. (2013a,b, 2014, 2015) highlighting the role of hydrological connectivity and habitat heterogeneity for microbial community composition and function in the hyporheic sediments (i.e., at the interface with groundwater) across various streams within proglacial floodplains. Understanding the spatiotemporal dynamics of microbial diversity across the streams that drain proglacial floodplains is critical as the environment of these ecosystems is changing owing to rapid glacier recession. In fact, it has been proposed that streams that are predominantly fed by glacier meltwaters today become increasingly fed by groundwater and snowmelt as glaciers shrink, with potentially major implications for their microbial communities (Freimann et al., 2013a; Wilhelm et al., 2013; Milner et al., 2017).

The aim of this study was to explore spatiotemporal patterns of microbial diversity associated with benthic biofilms across braided glacier-fed and tributary streams within three different proglacial floodplains in the Swiss Alps. Benthic biofilms typically dominate microbial life in streams (Battin et al., 2016), particularly in glacier-fed streams (e.g., Wilhelm et al., 2013), where they fulfill critical ecosystem processes. We hypothesized that both bacterial diversity and community structure differ between the braided glacier-fed system and its tributaries, essentially translating into a lateral chronosequence. Given the limited terrestrial subsidies of organic matter to proglacial streams, we also hypothesized that aquatic primary producers (e.g., algae) shape the bacterial communities of the benthic biofilms. We tested these hypotheses using 16S rRNA and 18S rRNA gene amplicon sequencing, and analyzing the resulting patterns of biodiversity, community composition and co-occurrences. Our findings unravel poorly-appreciated patterns of microbial diversity along chronosequences in proglacial floodplains and the role of benthic primary producers in shaping the bacterial communities.

## Material and methods

### Study sites and sample collection

**FIGURE 1:**
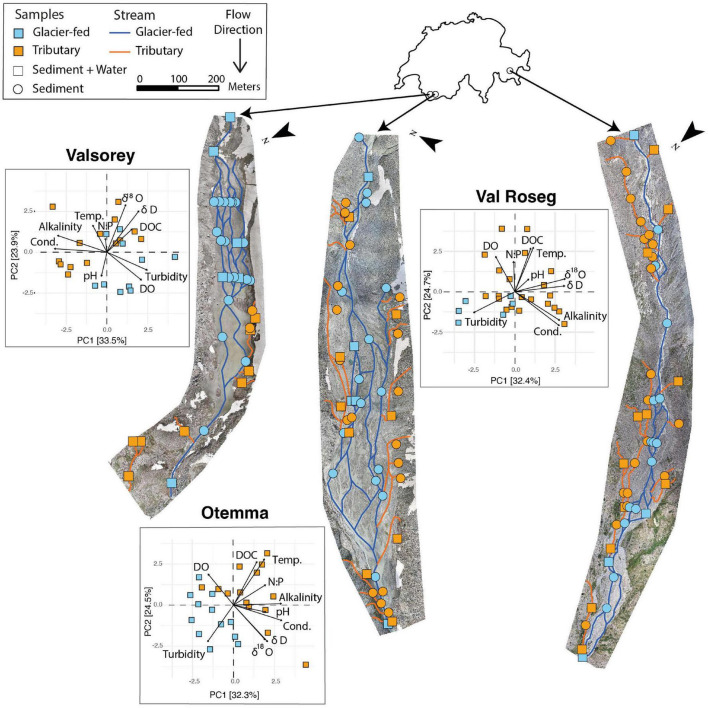
Maps of sampling locations for Valsorey, Otemma, and Val Roseg floodplains with principal component analysis on water samples. Streams were digitized from orthomosaics, with orange and blue lines as TRIB and GFSs, respectively. Round-shaped points identified sites with exclusive sediment sampling, while square-shaped points identified sites with both water and sediment sampling, providing a total of 131 benthic sediment and 40 water sites. Principal Component Analysis for individual floodplains using the water chemistry data collected for both seasons, where different colors represent the stream types (blue: GFS, orange: TRIB). The following acronyms were used to maximize space in the figure: Temp., Temperature; Cond., Conductivity. There is a separation between the GFS and TRIB samples mainly driven by temperature and conductivity.

We collected a total of 259 benthic sediment samples from glacier-fed streams (GFSs) and lateral tributaries (TRIBs) within the floodplains of the Otemma (OTE, 45° 56′ 08.4″ N 7° 24′ 55.1″ E), Val Roseg (VR, 46° 24′ 21.1″ N, 9° 51′ 55.1″ E), and Valsorey (SOY, 45° 55′ 09.4″ N, 7°15′ 34.2″ E) glaciers in the Swiss Alps. Study reaches ranged from the glacier snout to the outlet of the proglacial floodplain within terrain that has been ice-free for roughly 36 (OTE), 65 (VAR), and 70 years (SOY) (Figure 1). We geo-referenced each reach and sampled it once during June to July and later during August to September (2019). Floodplain characteristics and sampling information are detailed in Table 1. Within each reach, we randomly collected sandy sediment (0.250–3.15 mm) from the benthic zone (down to 5 cm in the streambed) using flame-sterilized sieves and spatulas. Samples (2.5–3 g) for DNA extraction, and the analysis of chlorophyll-*a* and extracellular polymeric substance (EPS), were immediately flash-frozen on dry ice in the field. Samples for bacterial abundance (BA) were preserved in 1.8 ml of filter-sterilized paraformaldehyde/glutaraldehyde solution prior to flash-freezing. Samples for the determination of bacterial carbon production (BCP), were also collected.

**TABLE 1:** Floodplain’s characteristics and sampling design.

	Otemma	Val Roseg	Valsorey
Altitude (m, a.s.l) (range)	2,400–2,600	2,090–2,340	2,400–2,500
Floodplain length (m)	1,300	1,100	1,000
Floodplain width (m) (range)	50–160	50–250	30–90
Early–sampling date	08–10 July	02–03 July	24–26 June
Early–#sample collected	50	44	34
GFS/TRIB	22/28	18/26	24/10
Late–sampling date	21–22 August	10–11 September	17–18 September
Late–#samples collected	49	50	34
GFS/TRIB	22/27	20/30	24/10
Total number of samples	97	94	68
GFS/TRIB	42/55	38/56	48/20

We collected streamwater samples from a subset of the GFS and TRIB study reaches. We measured streamwater temperature, pH, and dissolved oxygen using a WTW Multiparameter portable meter (MultiLine^®^ Multi 3630 IDS), conductivity using a WTW—IDS probe (TetraCon^®^ 925). Streamwater turbidity was monitored using PME Cyclops-7 loggers (every minute) and averaged over the duration of the sampling. Streamwater samples for the determination of dissolved organic carbon (DOC) were filtered (pre-combusted GF/F filters, Whatman, United Kingdom) and stored in acid-rinsed and pre-combusted glass vials. Aliquot samples for the determination of inorganic nutrients were collected in acid-washed 30 ml Nalgene HDPE bottles, flash-frozen on dry ice, and stored at −20°C. Samples for the analysis of major ions were filtered (0.22 μm filters Sterivex Millipore, Durapore PVDF membrane) and stored at 4°C.

### Streamwater analyses

The concentration of DOC was determined using a Sievers M5310c TOC Analyzer (GE Analytical Instruments) (accuracy: ±2%, precision: <1%, detection limit: 22 μg C/L), while inorganic nutrients (ammonium, nitrite, nitrate and soluble reactive phosphorus) were analyzed using a LaChat QuikChem 8500 flow injection analyzer.

### Bacterial abundance and chlorophyll-a

Bacterial abundance (cells per gram of dry sediment) was estimated using flow cytometry (Kohler et al., 2020a; Fodelianakis et al., 2021). Cell detachment was completed through mild shaking (Standard Analog Shaker, VWR, 15 min, 5.5 speed) and sonication (Sonifier 450, Branson, 1 min, 60% duty cycle, output 5) in 10 ml of paraformaldehyde/glutaraldehyde solution supplemented with sodium pyrophosphate (final concentration of 0.025 mM). The supernatant was diluted and stained with SybrGreen^®^ (1*x* final concentration, incubation for 15 min at 37°C) before analysis on a flow cytometer (NovoCyte, ACEA Biosciences) equipped with a 488 nm laser. For each sample, we analyzed three stained technical replicates and one unstained replicate. The coefficient of variation among technical replicates averaged 4.93 ± 3.84%.

Sediment chlorophyll-*a* concentration was measured following a modified ethanol extraction protocol (Kohler et al., 2020a). Briefly, ca. 2 g of wet sediment was mixed with 5 ml of 90% EtOH, placed in a hot water bath (78°C, 10 min), and incubated in the dark (4°C) for 24 h. Samples were then vortexed, centrifuged, and the supernatant read on a plate reader at 436/680 nm (excitation/emission). Concentrations of chlorophyll-*a* were quantified using spinach chlorophyll-*a* as a standard and are reported as μg chlorophyll-*a* g^–1^ DM.

### Extracellular polymeric substances

Extracellular polymeric substances was extracted from lyophilized sediments using 50 mM of ethylenediaminetetraacetic acid (EDTA) (1 h), followed by centrifugation at 4,200 rpm for 20 min at 4°C. The supernatant was sterile-filtered (0.2 μm) and ice-cold EtOH was added at a final concentration of 70%. Samples were then incubated at −20°C (24 h), after which EPS was repeatedly washed (70% EtOH) and pelleted. The final pellet was dried, re-suspended in 0.5 ml MQ water, and EPS measured as glucose equivalent according to Dubois et al. (1956).

### Bacterial carbon production

We measured BCP using [^3^H]-leucine (specific activity: 149.0 Ci/mmol, Perkin Elmer) incorporation in the field. Briefly, sediment samples (2.5–3 g) were incubated with 30 nM ^3^H-Leucine in the dark at *in situ* temperature (2 h), after which the incorporation was stopped by adding formaldehyde (3.7% final concentration). Killed controls were run in parallel. In the laboratory, radioactively labeled proteins were extracted and their label determined according to Fischer and Pusch (1999). Briefly, cells were detached from the sediments using vortexing and sonication (10 min, Sonorex super RK512, 35 KHz, 225/450 W, Bandelin), and proteins extracted by adding trichloroacetic acid (15% TCA). After incubation in a water bath (95°C, 30 min) samples were cooled on ice (30 min) to coagulate proteins. Next, proteins were collected onto 0.2 μm polycarbonate filters (Isopore, GTTP02500, Millipore, Merck) and rinsed twice with ice-cold 5% TCA and 4 times with MilliQ water. The samples were then transferred into scintillation vials containing 14 ml of scintillation cocktail (Optiphase Hisafe 3, Perkin Elmer) and disintegrations per minute (DPM) were measured (5 min) on a liquid scintillation counter (Tri-Carb 4910 TR, Perkin Elmer). DPM were converted to incorporated leucine using its specific activity, and bacterial protein production was calculated according to Simon and Azam (1989) and Marxsen (1996). From this, bacterial carbon production was calculated by multiplying the protein to cell carbon ratio (0.86).

### DNA extraction, metabarcoding library preparation, and sequencing

DNA was extracted using a modified phenol-chloroform method (Busi et al., 2020), scaled down to use 0.5 g of sediment inputs for accelerated sample processing. All DNA samples were diluted to a final concentration of ≤2–3 ng/μl. Prokaryotic metabarcoding libraries targeting the V3-V4 hypervariable region of the 16S rRNA gene with the 341F/785R primers were prepared following manufacturers guidelines. Eukaryotic 18S rRNA gene metabarcoding library preparation was performed likewise using the TAReuk454F-TAReukREV3 primers targeting the V4 loop (Stoeck et al., 2010). All amplifications were verified on a 1.5% agarose gel and a second PCR was realized to add dual indices to the purified amplicon PCR products, allowing for multiplexing of samples on a single sequencing lane of the MiSeq (Illumina) platform after quantification and normalization. Samples were sequenced using a 300-base paired-end protocol at the Lausanne Genomic Technologies Facility (LGTF, Switzerland).

### Bioinformatics

The 16S and 18S rRNA gene metabarcoding data were analyzed using a combination of Trimmomatic (Bolger et al., 2014) and QIIME2 (Bolyen et al., 2019) using the v138.1 SILVA database (Quast et al., 2013) for taxonomic classification. For the 16S rRNA amplicon dataset, a total of 257 amplicon sequence libraries were generated (four samples were discarded due to amplification failure) and paired-end sequencing generated a total of 32,186,859 reads, with an average of 124,273 reads per sample. Rarefaction curves for 16S rRNA and 18S rRNA showed saturation (Supplementary Figure 1). Non-bacterial Amplicon Sequence Variants (ASVs) including those affiliated to archaea, chloroplasts, and mitochondria were discarded from the 16S rRNA amplicon dataset. Singletons and ASVs observed only once were discarded. For the 18S rRNA amplicon dataset, 242 amplicon sequence libraries for sediment samples were generated (19 samples were discarded due to DNA extraction and amplification issues) and paired-end sequencing generated a total of 21,413,150 reads. The resulting 18S ASVs were clustered into operational taxonomic units (OTUs) using a 97% identity threshold using the vsearch *de novo* clustering method implemented in QIIME2 to avoid overestimation of diversity by the high copy number of 18S in the cells. Given the uncertainty regarding sampling, amplification, and sequencing of larger eukaryotes, non-phototrophic eukaryotes were discarded from the 18S rRNA amplicon dataset in all downstream analyses (except for the co-occurrence network analysis where fungi were included). Singletons and OTUs observed only in one sample were discarded from the 18S rRNA amplicon dataset in all downstream analyses, resulting in an 18S rRNA phototrophs dataset with 429 OTUs.

### Statistical analyses

All statistical analyses were performed in *R* 4.0.3 (R Core Team, 2021). We used a permutational analysis of variance (PERMANOVA) on the Euclidean distance between sites to evaluate the effects of proglacial floodplains (OTE, VAR, and SOY), stream type (GFS versus TRIB), and season (early versus late) on the physicochemical characteristics of the streamwater, using the *adonis* function in the *vegan* package (Oksanen et al., 2020). To visualize differences in environmental variables, we performed a Principal Components Analysis (PCA), using *factoextra:fviz_pca_biplot* (Kassambara and Mundt, 2020), to visualize the effects of environmental variables on the separation of GFSs and TRIBS. Differences between proglacial floodplain, stream type, and season in biofilm BA, chlorophyll-*a*, EPS, and BCP were assessed using three-way ANOVAs from *stats* R package (R Core Team, 2021). We used *t*-tests to assess the difference between stream type for each biomass indicator and glacier floodplain; *p*-values were adjusted following Benjamini and Hochberg (1995).

We computed non-metric multidimensional scaling (NMDS) ordinations based on Bray-Curtis dissimilarity distances (including 999 permutations) for both 16S and 18S datasets with the *vegan* R package to illustrate differences in microbial communities as a function of stream type and glacier floodplain. To highlight the role of phototrophs in structuring bacterial communities, we projected the first axis of the 18S rRNA NMDS onto the bacterial NMDS using *vegan:ordisurf*. We also performed a procrustes analysis using *vegan:procrustes* to assess the correlation between 16S and 18S datasets. We tested the effects of stream type on bacterial compositional variability using *t*-test and analysis of multivariate homogeneity of group dispersions on the Bray-Curtis dissimilarity matrices using the function *vegan*:*betadisper*. A PERMANOVA on Bray-Curtis dissimilarity matrix was used to test the effects of stream type, glacier floodplain, season, on bacterial community composition with the function *vegan:adonis*. For each glacier floodplain, differences in community similarity of the two stream types were assessed using *pairwise.adonis* (Martinez Arbizu, 2020), and *p*-values were adjusted according to the Benjamini and Hochberg method. Additionally, we included chlorophyll-*a* as a proxy for phototrophic activity as an additional effect on 16S Bray-Curtis dissimilarity in PERMANOVA.

For alpha diversity, we estimated 16S ASV and 18S OTU richness using *breakaway* (Willis and Bunge, 2015) as well as Pielou’s evenness, and tested for significance differences between stream type using *breakaway:betta* (Willis et al., 2017). The percentage of ASVs only present in glacier-fed streams, tributary streams, and the shared portion between the two stream types were computed using the function *MicEco*:*ps_venn* (Russel, 2021). The analysis was performed for each floodplain separately and computed for both presence/absence and abundance-weighted data. We next analyzed presence/absence patterns to identify ASVs with a high potential to be lost as glacier-fed streams disappear. We used Fisher’s tests of odds ratios to evaluate the likelihood of individual ASVS to be exclusively present in glacier-fed streams. We deem ASVs with a significant (adjusting *p*-values with the BH method) high likelihood (odds ratio > 1) of being present in glacier-fed streams and absent in tributary streams to be at high risk once glacier-fed streams disappear. Finally, we identified genera which were enriched in ASVs likely to be present only in GFSs using Fisher’s tests. After discarding non-significant adjusted *p*-values, 25 genera were identified.

We constructed co-occurrence networks based on samples for which both 16S and 18S rRNA gene amplicons were available, that were sequenced during both seasons, and that had a consistent categorization across seasons. This resulted in a total of 198 samples, with 92 and 106 samples for GFS and TRIB, respectively. All ASVs present in less than 5% of the samples were discarded from the 16S dataset (Peschel et al., 2021). Co-occurrence networks between 16S and 18S rRNA gene amplicons (retaining photoautotrophs and fungi only) were constructed using an average of the distance matrices created from SparCC, Spearman’s correlation, and SpiecEasi where the networks were constructed using the Meinshausen and Bühlmann (mb) method (Meinshausen and Bühlmann, 2006). This provided one consensus network for GFS and one for TRIB. Negative edges were removed to visualize only mutualistic relationships and edges were further filtered to keep exclusively prokaryote—eukaryote interactions. Additionally, we used stringent cutoffs to avoid spurious correlations and kept only the top 10% edges in terms of their interaction strength. To detect clusters within the networks, we used the fast-greedy clustering algorithm (Clauset et al., 2004; Newman, 2004) and removed clusters with less than 5 nodes. We then calculated network topology measures, including node and edge number, number of clusters, diameter and modularity. The networks were visualized using the *igraph* R package (Csardi and Nepusz, 2006).

## Results and discussion

### Diverse stream habitats across proglacial floodplains

Depending on channel stability and water sources, the physicochemical template of streams can vary significantly across a proglacial floodplain (Milner and Petts, 1994; Milner et al., 2010, 2017). To describe this template, we measured streamwater temperature, electrical conductivity, turbidity, pH, alkalinity, water stable isotopes, and both DOC and inorganic nutrient concentrations in GFSs and TRIBs across the three floodplains (Figure 1 and Supplementary Table 1). Using PCA, we found that in all three floodplains, physicochemical characteristics markedly differed between GFSs and TRIBs (Figure 1), highlighting environmental gradients along the lateral chronosequence. Consistent with previous work (Brown et al., 2003; Milner et al., 2010; Uehlinger et al., 2010; Robinson et al., 2016), streamwater temperature, DOC concentration, and electrical conductivity were significantly higher in TRIBs, while turbidity was higher in GFSs but negligible in TRIBs (ANOVA, *p* < 0.001 for all, Supplementary Tables 1, 2). The concentration of dissolved inorganic nitrogen (DIN) was higher in GFSs than in TRIBs (ANOVA, *p* < 0.001), which may be attributable to DIN release from ice and snowpack where it has accumulated over winter (e.g., Tockner et al., 2002); snow-pack related DIN sources to TRIBS may have depleted already at the time of sampling. Similarly, soluble reactive phosphorus (SRP) concentrations were on average 3.2 times higher in GFSs than in TRIBs (ANOVA, *p* < 0.001, Supplementary Tables 1, 2). This may be attributable to glacial weathering processes that, depending on geology, can produce large amounts of soluble and particulate P (Hodson et al., 2004; Dubnick et al., 2020). We observed neither a seasonal effect on the physicochemical properties in GFSs and TRIBs, nor marked downstream gradients along the longitudinal chronosequence (Supplementary Tables 2, 3).

### Biofilm biomass and bacterial carbon production differ between stream types

We found benthic chlorophyll-*a*, serving as a proxy for algal biomass, to be 35 times higher on average in TRIBs than GFSs (Table 2 and Supplementary Table 4). We attribute this to differences in channel stability, discharge (both not shown here) and turbidity in GFSs and TRIBs. Elevated turbidity attenuates light, thereby reducing primary production (Uehlinger et al., 2010; Boix et al., 2021), while suspended sediments can abrase benthic algae (Francoeur and Biggs, 2006). Similar to chlorophyll-*a*, bacterial abundance was ten times greater on average in TRIBs than in GFSs (Table 2). We tentatively ascribe this effect to the benthic algae, which are a major energy source to bacteria in streams in general (Kaplan and Bott, 1989; Wagner et al., 2017) and particularly in streams devoid of major terrestrial subsidies of organic matter such as those in proglacial floodplains.

**TABLE 2:** Biomass indicators for each glacier floodplain and stream type (mean ± standard deviation).

	Otemma			Val Roseg			Valsorey		
	GFS	Tributary	*P*-value	GFS	Tributary	*P*-value	GFS	Tributary	*P*-value
Chl-*a* [ug g^–1^_*DM*_]	0.02 ± 0.06	0.37 ± 0.59	**<0.001**	0.02 ± 0.05	0.66 ± 1.38	**0.002**	0.00 ± 0.01	0.80 ± 1.01	**0.002**
Bacterial Abundance [10^6^ cells g^–1^_*DM*_]	2.94 ± 1.85	30.9 ± 49.3	**<0.001**	3.67 ± 2.86	30.0 ± 49.6	**<0.001**	5.38 ± 4.91	89.9 ± 63.9	**<0.001**
Bacterial Carbon Production [10^–3^ ngC g^–1^_*DM*_ h^–1^]	1.85 ± 1.87	3.89 ± 3.27	**<0.001**	2.46 ± 1.16	4.37 ± 3.06	**<0.001**	1.44 ± 0.92	1.76 ± 1.45	0.36
Extracellular Polymeric Substance[glucose-equivalent g^–1^_*DM*_]	0.63 ± 1.27	1.26 ± 1.73	**0.04**	0.26 ± 0.67	1.35 ± 3	**0.017**	13.5 ± 20	22.2 ± 22.9	0.14

A Welch Two Sample t-test was used to test the significant difference between GFS and TRIB and significant P-values are reported in bold. DM, Dry mass of sediment.

We also found higher amounts of EPS associated with the biofilms in TRIBs than GFSs, which may reflect overall biomass patterns in these two stream types. However, when normalizing EPS by cell abundance, the pattern inversed (GFS: 1.62 ± 4.31 × 10^–6^ glucose-equivalent cell^–1^; TRIB: 0.118 ± 0.288 × 10^–6^ glucose-equivalent cell^–1^) (ANOVA, *F* = 6.246, *p* < 0.05). Higher EPS per cell was previously reported as a mechanism to withstand flow-induced erosion in GFSs (Battin et al., 2001) and sequester organic compounds to overcome starvation in DOC-poor stream ecosystems (Freeman and Lock, 1995). Finally, we found higher average BCP in TRIBs than in GFSs, possibly also a result of higher cell numbers in TRIBs. However, specific BCP (normalized by bacterial cell abundance) was significantly (ANOVA, *F* = 14.530, *p* < 0.001) higher in GFSs (0.767 ± 0.893 × 10^–9^ ng C cell^–1^ h^–1^) than TRIBs (0.499 ± 0.788 × 10^–9^ ng C cell^–1^ h^–1^) (Supplementary Figure 2).

Our findings suggest that benthic biofilms in TRIBs are more productive and accumulate more biomass than in GFSs. This may be related to the overall higher environmental stability in TRIBs, specifically in those that drain slightly elevated terrasses at the edge of the proglacial floodplains and are disconnected from the GFS mainstem.

### Spatial patterns of microbial diversity between stream types

**FIGURE 2:**
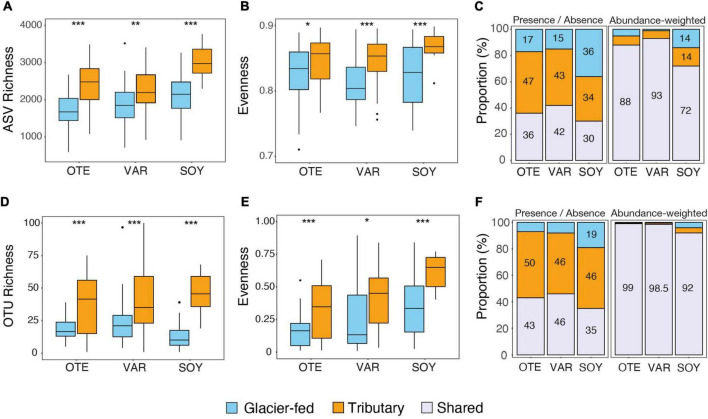
Alpha Diversity for bacterial (16S) and phototrophic (18S) communities. Boxplots summarized for each glacier floodplain and stream category **(A)** Amplicon Sequence Variants (ASV) richness, **(B)** Pielou’s evenness for bacterial communities, **(D)** 18S rRNA OTUs richness, and **(E)** Pielou’s evenness for phototrophic communities. Box plots show the median (horizontal line), interquartile range (box height), 1.5× beyond the interquartile range (whiskers), and outliers. A two-sample *t*-test established a significant difference between tributaries and GFSs for all three floodplains. **(C)** Bar plots summarized the proportion of **(C)** bacterial ASVs and **(F)** phototrophic OTUs exclusively present in GFS, TRIB, and the shared between these two stream types. Input for unweighted bar plots was presence/absence, while abundance-weighted bar plots used ASV abundance.

The benthic biofilms in both GFSs and TRIBs within the three proglacial floodplains contained diverse microbial communities. Overall, we detected a total of 53,858 16S rRNA ASVs and estimated richness was significantly greater in TRIBs compared to GFSs (*betta*, *p* < 0.01) with an average value of 2,461 ± 626 ASVs per sample in TRIBs compared to 1,921 ± 509 in GFSs (Figure 2A). This difference in alpha diversity between stream types was similar for all three floodplains and both seasons. Moreover, we identified 3,553 18S rRNA OTUs, highlighting the large diversity of eukaryotes in proglacial streams (Supplementary Figure 3). Phototrophic eukaryotes (i.e., microalgae classified as Chlorophyta, Charophyta, Cryptomonadales, and Ochrophyta) accounted for 429 18S rRNA OTUs, and for 62.2% of the eukaryotic relative abundance on average. We found significantly higher alpha diversity of phototrophic eukaryotes in TRIBs than in GFSs across all three proglacial floodplains (Figures 2D,E). A comparable difference in diversity between glacier-fed and groundwater-fed streams was previously reported for Rocky Mountain streams and attributed to increased streamwater temperature, reduced flow variability, and increased streambed stability (Hotaling et al., 2019).

While estimated richness based on 16S rRNA differed significantly between stream types, evenness (Pielou’s) did not differ between GFS and TRIBs across all floodplains (Figure 2B). However, we found significantly fewer rare taxa (with relative abundance < 0.01%) in GFSs (OTE: 3.16 ± 1.37%; VAR: 4.19 ± 1.75%; SOY: 4.61 ± 1.68%) than in TRIBs (OTE: 5.26 ± 1.77%; VAR: 4.81 ± 2.03%; SOY: 6.44 ± 1.34%) (Welch two-sample *t*-test, *p* < 0.001) (Supplementary Figure 4). This is striking because it indicates a preferential exclusion of rare taxa in GFSs. Rare microbial taxa are thought to be dormant or metabolically inactive and may transiently pass through an environment or persist as conditionally rare taxa (Newton and Shade, 2016). This persistence of rare taxa is linked to the time-scale of stability of the environment. The low sediment stability in GFSs may limit the capacity of metabolically inactive taxa to persist. Consequently, one might expect few but metabolically active rare taxa in GFS. In line with this, the rare biosphere of glacier-fed streams has been previously shown to harbor a disproportionately large number of metabolically active taxa (Wilhelm et al., 2014) which can act as a seedbank and are thus potentially important to sustaining biodiversity in GFSs. Moreover, we did not observe pronounced re-arrangement in the relative abundance of taxa between the two sampling periods (Supplementary Figure 5). Instead, the relative abundance of taxa remained remarkably conserved between the first and second sampling in both TRIBs and GFS. This contrasts with the notion of conditionally rare taxa (Shade et al., 2014), which refers to transient states of rarity and abundance. While our sampling design clearly did not resolve the full range of temporal variability possible, we tentatively attribute the deprivation of rare taxa in GFSs to their small population sizes, rendering them prone to stochastic extinction related to the particularly unstable sedimentary environment in these habitats. Rare and metabolically inactive taxa, even conditionally rare taxa, may be constantly lost from GFS. On the other hand, the environment of TRIBs is more stable and diverse niches may develop within the copious biofilms (e.g., higher biomass), thus increasing the probability of rare taxa to persist over longer time scales than in GFSs.

Moreover, we observed a significant, yet gradual, increase in prokaryotic richness along the longitudinal gradient of the GFS from the glacier snout to the outlet of OTE and SOY (Supplementary Figure 6). While chronosequences on proglacial streams are an imperfect space-for-time substitution (Ficetola, 2021), mostly due to the directional connectivity of GFSs, similar patterns have been observed for soil microbial diversity (Franzetti et al., 2020) and plant-insect studies (Albrecht, 2010). We posit that environmental factors (e.g., increasing sediment stability and water temperature) and dispersal from tributary streams account for the increase in diversity along the longitudinal gradient.

To further unravel patterns of diversity within and among stream types in proglacial floodplains, we quantified the presence and absence of taxa across the two stream types. For individual floodplains, the proportion of 16S ASVs unique to TRIBs ranged from 34 to 47%, and from 36 to 15% for GFSs (Figure 2C), highlighting that a considerable fraction of biodiversity was specific to either stream type. Similarly, the proportion of 18S OTUs unique to TRIBs ranged from 46 to 50%, and from 7 to 19% for GFSs (Figure 2F), highlighting the large fraction of phototrophic eukaryotes diversity in TRIBs. This is particularly important in light of biodiversity loss as glaciers continue to vanish and proglacial floodplains will be increasingly dominated by groundwater-fed streams. However, when accounting for abundance, we found that prokaryotic ASVs shared between stream types accounted for 72–93% of relative abundance in the three glacial floodplains (Figure 2C). Hence, abundant taxa appear in both stream types, indicating that common environmental drivers, such as the low water temperature, oligotrophy and high UV radiation shape the metacommunity of proglacial floodplain stream ecosystems.

Next, we deciphered the taxonomy underlying the microbial diversity observed in GFSs and TRIBs. Overall, we found that prokaryotic diversity comprised 55 bacterial phyla, 162 classes, 402 orders, 599 families, and 1,061 genera. The most diverse and abundant phyla were Proteobacteria (13,503 ASVs; accounting for 31.1–87.9% of relative abundance), followed by Bacteriodota (6,771 ASVs; 1.9–32.6%), and Planctomycetota (5,977 ASVs; 0.4–17.1%). Proteobacteria and Bacteriodota are known to be abundant in glacier environments, probably due to their ability to thrive in oligotrophic environments and degrade organic substances (Boetius et al., 2015; Anesio et al., 2017). To further evaluate differences in taxonomic composition between GFSs and TRIBs, we compared the relative abundance of the most abundant prokaryotic groups. Consistently across the three glacial floodplains, we found Alphaproteobacteria, Bacteroidia, Planctomycetes, Verrucomicrobiae, and Cyanobacteria to be significantly more abundant in TRIBs than GFSs, while Gammaproteobacteria were more abundant in GFSs (Supplementary Figure 7).

Next, we used binomial models and odds ratios to quantify the likelihood of particular genera present in GFSs only. These genera may be the most vulnerable to climate-induced changes of the GFS environment. *Caenimonas*, *Nitrotoga*, *KDA-96*, *Leeia*, *Nitrospira*, *Nitrosospira*, *Polaromonas*, *Rhodoferax*, and *Thiobacillus* were some of the genera identified with the highest odds ratios (Supplementary Figure 8). *Polaromonas* is a widespread, facultatively chemolithotrophic bacterium dwelling in cryospheric systems (Darcy et al., 2011; Smith and Foreman, 2014). *Nitrotoga* and *Nitrospira* are nitrite oxidizers and have been reported from glacier meltwater (Kohler et al., 2020b) and cold-water rivers (Liu et al., 2020). The sulfur-oxidizing and chemolithotroph *Thiobacillus* is often found in cold-related environments (Kohler et al., 2020b; Fodelianakis et al., 2021) and is also known for its genomic cold adaptation (Harrold et al., 2016). Hence, bacteria identified to be most vulnerable to changes of the GFS environment are involved in relevant biogeochemical pathways with potential downstream impacts for the flux of nutrients and trace elements.

Exploring the eukaryotic photoautotroph diversity, we observed that Ochrophytes were generally dominant, accounting for 54.5% of all reads on average across samples, and ranging from 20.1% in VAR TRIBs to 75.1% in OTE GFSs (Supplementary Figure 3). This is consistent with results found in other alpine streams (Uehlinger et al., 1998; Hieber et al., 2001; Rott et al., 2006; Robinson et al., 2010). Specifically, *Hydrurus foetidus*, a psychrophilic golden alga often found in cold fast-flowing alpine streams (Klaveness, 2019), accounted on average for 69.5% of the reads classified as Ochrophyta. Bacillariophyceae accounted for another 26.0% on average. We identified 28 different genera of Bacillariophyceae, with *Achnanthidium*, *Cocconeis*, *Cymbella*, *Navicula* and *Reimeria* being the most abundant. Chlorophyta (average 3.6%) and Charophyta (average 2.7%) were other important phototrophic community members. Bacillariophyceae were significantly more abundant in TRIBs, accounting for 30.2% of the relative abundance on average compared to 5% in GFS samples (Supplementary Figure 9). Chrysophyceae accounted for 82% of relative abundance in GFSs on average and for 55% in TRIBs (Supplementary Figure 9).

Overall, our findings reveal TRIBs as ecosystems with an elevated taxonomic diversity of prokaryotes and eukaryotic photoautotrophs, alongside elevated microbial biomass and activity (i.e., BCP), within proglacial floodplains. We suggest that this pattern is associated with the environment that becomes relatively more stable along the lateral chronosequence—from the braided GFS to the TRIBs that drain the edge of the floodplains (Figure 1). This pattern is in line with the general notion of ecological succession and biodiversity along chronosequences within glacial forelands (Ficetola, 2021).

### Community structure differs between stream types

**FIGURE 3:**
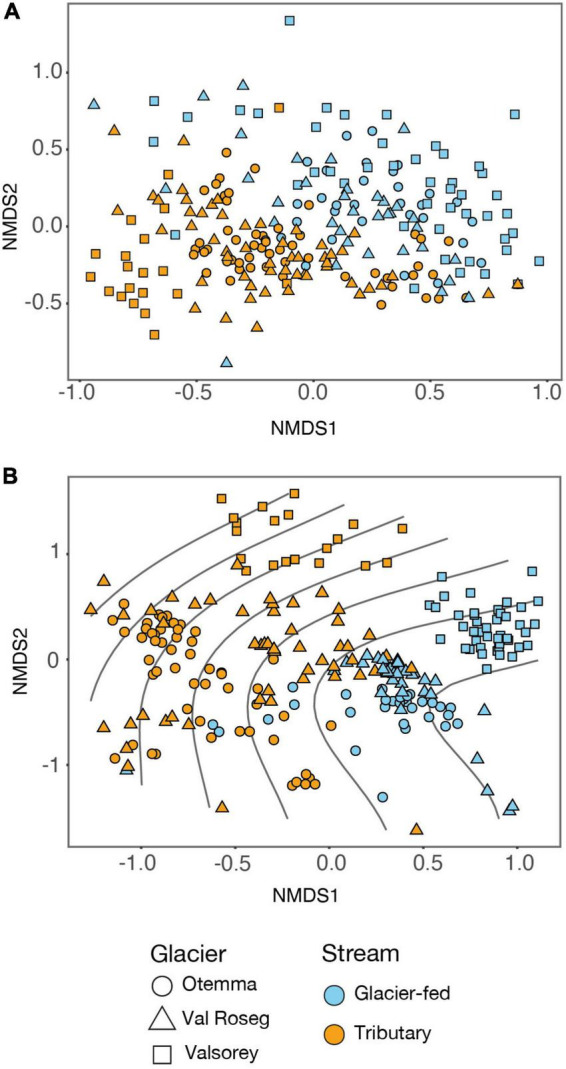
Patterns of bacterial community structure in glacier floodplains. Sample ordination is a non-metric multidimensional scaling (NMDS) based on Bray-Curtis dissimilarity for **(A)** 18S rRNA (stress value: 0.24) and **(B)** 16S rRNA (stress value: 0.19). Colors indicate the two-stream types, and symbol shapes specify the three glacier floodplains. Smooth response curves in panel B were obtained from site scores (first NMDS axis) of phototrophic 18S NMDS and indicate that the differences in prokaryotic community similarity between stream types were also associated with changes in phototrophic eukaryotic community similarities.

Using unconstrained ordination analysis, we found that prokaryotic communities clustered by stream type and floodplain (Figure 3B), while phototrophic eukaryotes separated mostly by stream type (Figure 3A). Strikingly, the differences in prokaryotic community similarity between stream types were associated with changes in phototrophic eukaryote community similarities (Figure 3). The procrustes analysis indicated a significant (*p* < 0.001) procrustes correlation (0.43) between phototrophic and prokaryotic communities. We suggest this as evidence toward phototroph-heterotroph coupling which structures stream biofilm communities in proglacial floodplains. This observation is further corroborated by the role benthic chlorophyll-*a* plays besides stream type and floodplain by explaining 2.1%, 9.7% and 9.3%, respectively, of the variance in prokaryotic Bray-Curtis dissimilarity (BC). In comparison, seasonality only explained 0.6% of variance in prokaryotic community similarity (Supplementary Table 5).

Using the distances to the centroid in NMDS as a measure of beta diversity, we assessed the variability of community composition among GFSs and TRIBs. Across the three glacial floodplains, GFSs had significantly lower distances to the centroids than TRIBs (Supplementary Figure 10). On the one hand, we attribute this pattern to different degrees of hydrological connectivity, which is arguably high within the GFS braided system but low between TRIBs. On the other, the relative contribution of groundwater and snowmelt to the flow in TRIBs differs depending on the location within the floodplain, ultimately resulting in elevated environmental heterogeneity among them. Furthermore, the rich communities of photoautotrophs varying across TRIBs may further affect the observed beta diversity of the prokaryotic communities.

In all three proglacial floodplains, we found significant longitudinal changes in community similarity from the GFSs close to the glacier snout to the outlet of the floodplain (Supplementary Figure 11). These patterns are unexpected given the relatively short distances (hundreds of meters) along the longitudinal chronosequence and the high dispersal of microbial cells in GFSs (Ezzat et al., 2022). We attribute this spatial turnover to downstream changes in the GFS environment that may be partially induced by processes (e.g., sedimentary dynamics) in the GFS channel itself, but also by the cumulative influence of the tributaries discharging into the GFS.

### Co-occurring patterns of prokaryotes and eukaryotes

**FIGURE 4:**
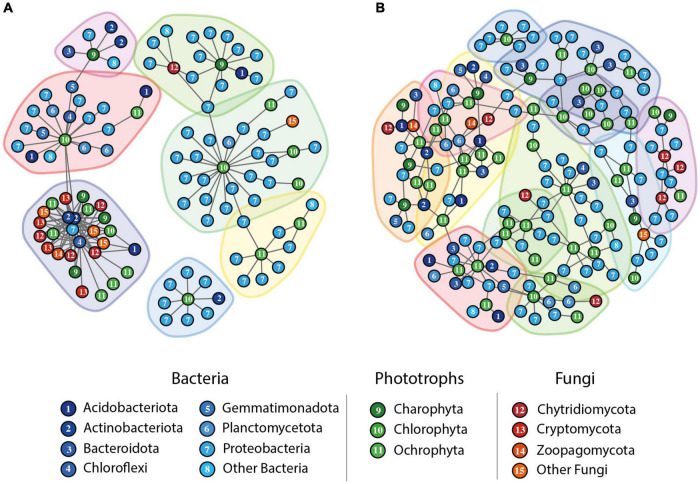
Co-occurrence Networks between prokaryotic and eukaryotic organisms. Only the top 10% positive interactions between prokaryotic and eukaryotic (phototrophs + fungi) organisms are displayed. **(A)** GFS network with *n* = 92 GFS sites and **(B)** TRIB network with *n* = 106 tributary samples. Clusters were computed using the fast-greedy algorithm and all clusters with less than 5 nodes were removed. Colors differentiate bacteria (blue), phototrophs (green), and fungi (red) and number provides information on the nodes’ specific taxonomy.

Our findings on community composition suggest a certain role of photoautotrophs in structuring prokaryotic communities. Therefore, to further explore the relationships between photoautotrophs and prokaryotes we analyzed co-occurrence networks and focused on positive interactions. Among the eukaryotes, we also included fungi, which (particularly Chytridiomycota) may parasitize on algae. We found that the GFS co-occurrence network had fewer nodes and edges, and shorter diameters, compared to the TRIB network (Table 3). Consistent with our results on diversity, photoautotrophs represented 29.3% of nodes in TRIB networks, compared to 18% in the GFS network. Bacterial and fungal taxa contributed to 69% and 12% of the nodes in GFSs, respectively, and 64.7% and 6%, respectively, in TRIBs (Table 3). The fast-greedy algorithm identified 7 and 12 clusters in the GFS and TRIB co-occurrence network, respectively (Table 3 and Figure 4),

**TABLE 3:** Co-occurrence network topology indices.

	GFS	TRIB
# Edges	165	205
# Nodes	113	167
% Bacteria	69%	64.7%
% Phototrophs	18%	29.3%
% Fungi	12%	6.0%
Diameter	2.81	9.83
Mean distance	3.36	9.59
# Clusters	7	12
Modularity	0.72	0.78

Interestingly, we found photoautotrophic nodes to be consistently located at the center of prokaryotic clusters in TRIBs (Figure 4), highlighting their roles in structuring the biofilm communities in these streams. Members of Charophyta, Chlorophyta, and Ochrophyta were represented in both networks but with different relative abundances. For instance, in the TRIB network, Diatomea represented 47% of all photoautotrophs (Supplementary Figure 12B), compared to 33% in the GFS network. Additionally, Ulvophycae, Xanthophyceae, and other photoautotrophic taxa were present exclusively in the TRIB network. Such apparent photoautotrophic-prokaryotic relationships may be metabolic in nature, as algal exudates have been reported to be an important source of organic matter to microbial heterotrophs in biofilms (e.g., Kaplan and Bott, 1989; Wagner et al., 2017). This may be particularly relevant in proglacial streams, which are largely devoid of terrestrially-derived organic matter. In contrast, prokaryotic nodes were rather located at the peripheries of these clusters in both co-occurrence networks, suggesting that individual photoautotrophs may engage in several interactions with various prokaryotes, whereas individual prokaryotes may instead engage in a single interaction with a phototrophic eukaryote.

Our network analysis also revealed the relevance of Chytridiomycota, which was involved in 12.8% and 11% of the interactions in the GFS and TRIB networks, respectively. Indeed, this is in line with their taxonomic dominance in TRIBs, whereas both Cryptomycota and Chytridiomycota dominated in GFSs (Supplementary Figure 12C). We also found that interactions between Chytridiomycota and photoautotrophs represented 41% and 33% of all interactions involving fungi in GFS and TRIB networks, respectively, while interactions between Chytridiomycota and bacteria accounted for 36% and 48%, respectively. This network pattern points to a potential role of Chytridiomycota in structuring biofilm communities in proglacial streams. In fact, Chytridiomycota have received increasing scientific interest as common microparasites of marine and freshwater algae (Grossart et al., 2019; Klawonn et al., 2021) as well as in the cryosphere (Brown et al., 2015; Lutz et al., 2015; Anesio et al., 2017; Rojas-Jimenez et al., 2017; Kohler et al., 2022) where they may shunt algal carbon to fungal biomass, thereby bypassing the typical microbial loop. Given that vascular plant debris is typically absent in proglacial streams, fungi parasitizing on benthic algae seems intuitive.

## Conclusion

Our findings highlight the high diversity of prokaryotes and eukaryotic photoautotrophs in streams draining proglacial floodplains. We attribute these biodiversity patterns to the environmental heterogeneity that develops along the chronosequences (particularly the lateral ones) in proglacial floodplains despite their relatively small spatial scale. The dynamics of tributaries draining fluvial terrasses is disconnected from glacier runoff dynamics, making tributary habitats more stable compared to the braided glacier-fed streams. As a consequence, benthic biofilms in tributaries can build up more photoautotrophic biomass, which putatively translates into niche diversification and provides highly available organic matter to the bacterial heterotrophs. The more subtle differences along the longitudinal chronosequence are striking because they both indicate the efficient colonization of sediment bed in newly deglaciated reaches, and that with significant lateral input of biodiversity the environmental conditions in glacier-fed streams support substantial microbial biodiversity.

## Data availability statement

The data presented in this study are deposited in the Zenodo repository, accession number: 6424496 (https://doi.org/10.5281/zenodo.6424496). Sequencing data have been uploaded to the National Center for Biotechnology Information under accession number PRJNA808857. R code underlying statistical analyses and models is available on GitHub (https://github.com/jadebrandani/Proglacial_floodplain_diversity).

## Author contributions

JB, TK, SF, HP, TB, and SL conceived and designed the study. JB, TK, SF, HP, and MR performed sampling. MR and SL provided orthomosaic data. JB, HP, TK, PP, SB, and SF performed lab work. JB, SF, MB, and SB analyzed the sequencing data. TB and SL provided resources. JB, HP, TK, and TB wrote the manuscript with input from SB, SF, LE, GM, MB, PP, MR, and SL. All authors contributed to the article and approved the submitted version.
