# Supplementary material for: Spatial patterns of benthic biofilm diversity among streams draining proglacial floodplains
Source: Front Microbiol. 2022 Aug 8;13:948165. doi: 10.3389/fmicb.2022.948165 (PMC9393633; doi:10.3389/fmicb.2022.948165)
Supplement: Supplementary file 1 [file Data_Sheet_1.pdf]

Supplementary Material

1 Supplementary Figures

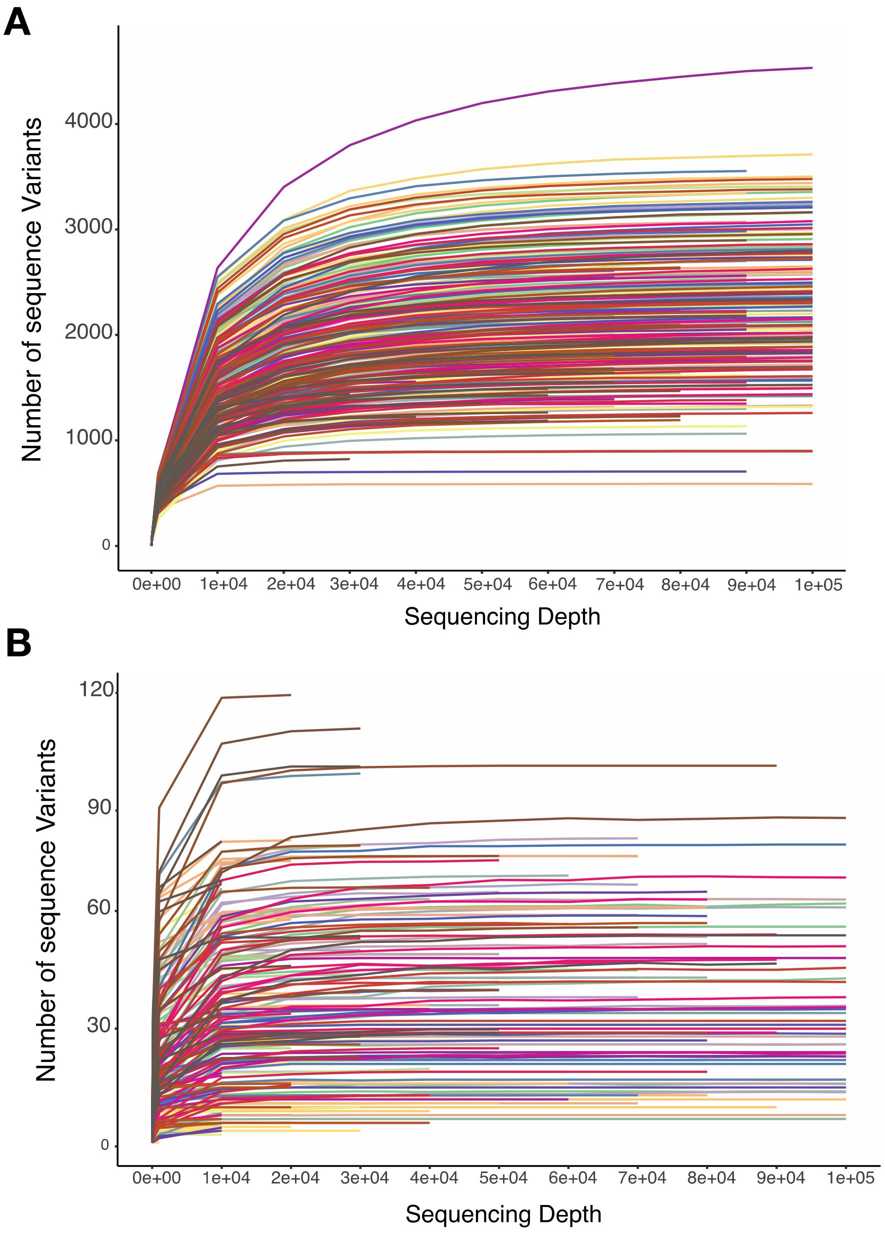

Figure S1. Rarefaction curves of 16S rRNA (A) and 18S (B) gene amplicon from all the sediment samples. The number of sequences is displayed on the x-axis and the number of observed sequence variants (ASVs) is shown on the y-axis. Rarefaction was performed

at intervals of 100 sequences with 10 permutations per interval at a sequencing depth up to 100’000 reads. Different colors represent the different samples n_{16S}= 259 n_{18S}= 242.

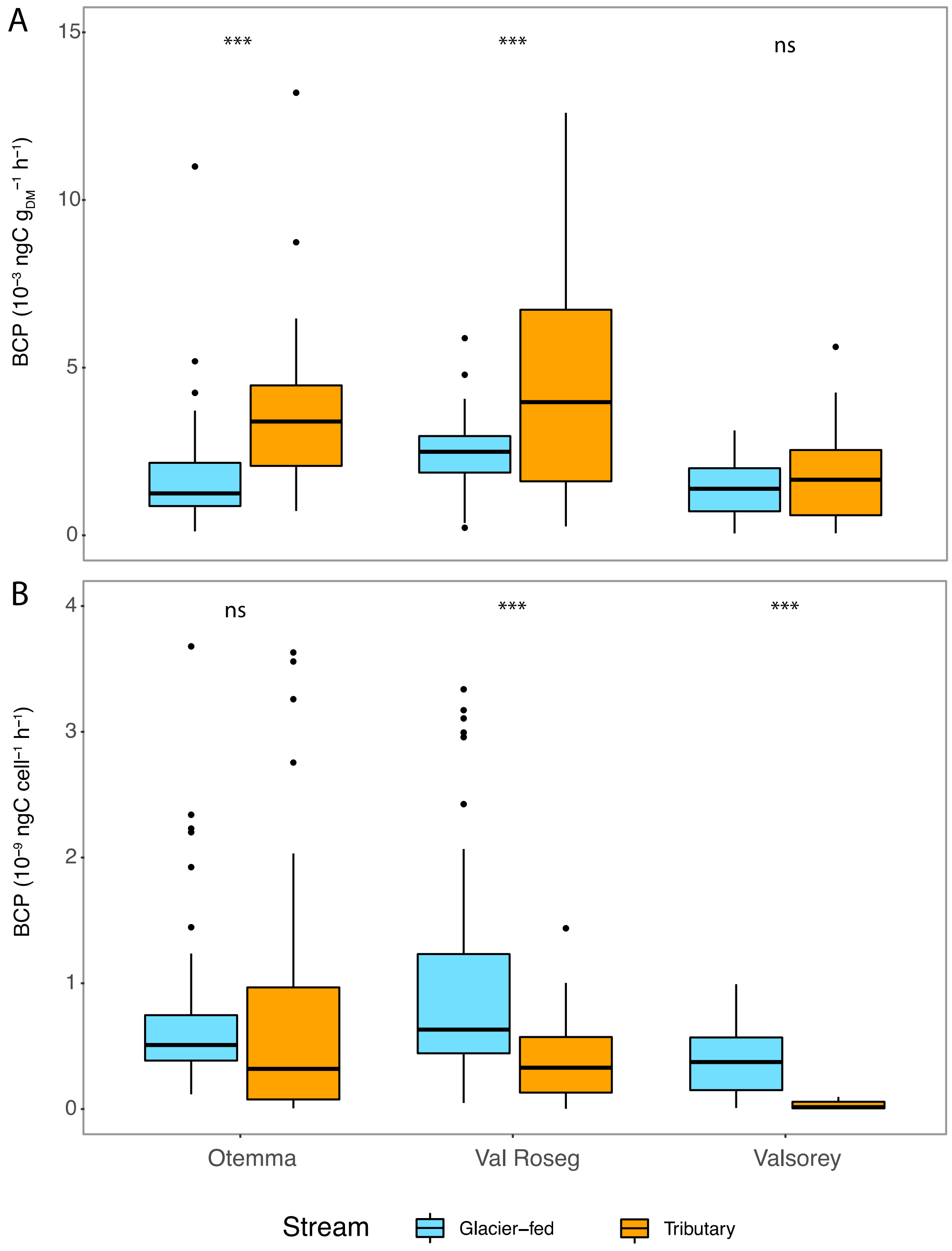

Figure S2. Boxplots displaying the difference in bacterial carbon production (BCP) for GFSs and TRIBs for the three glacial floodplains with (A) per gram of dry mass and (B) normalized by cell count. Boxes show the median (horizontal line), interquartile range (box height), 1.5 x beyond the interquartile range (whiskers), and outliers.

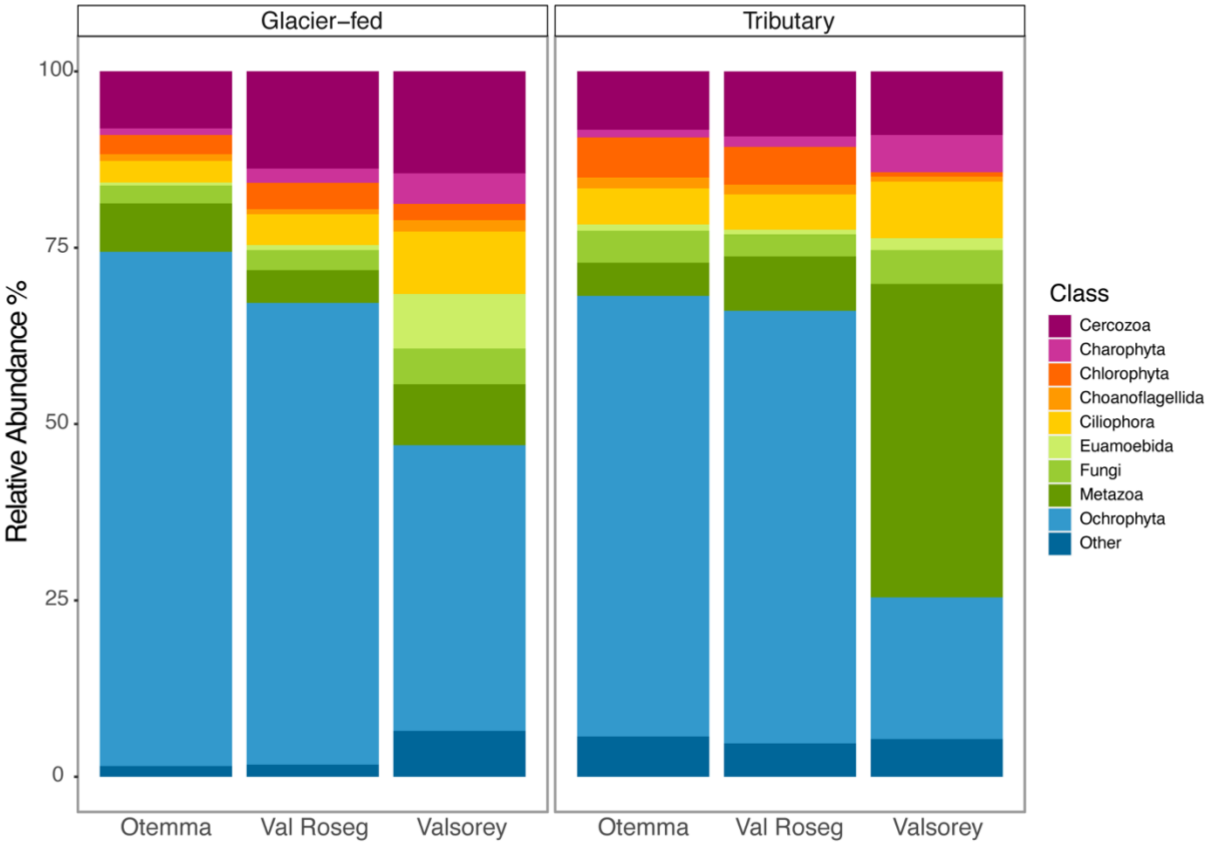

Figure S3. Barplots displaying the relative proportion of 18S rRNA gene amplification at the class level for each glacier floodplain and both stream types.

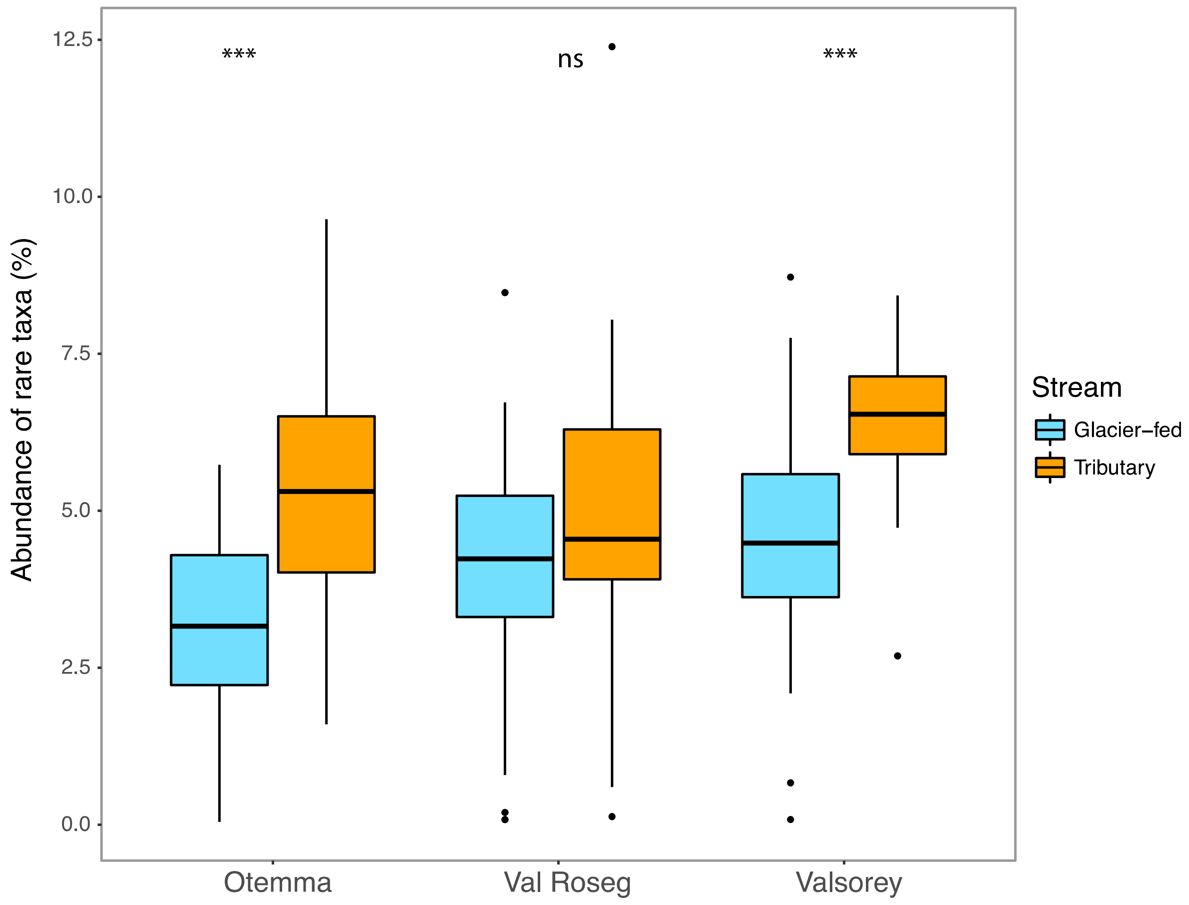

Figure S4. Boxplots displaying the abundance of rare taxa (abundance <0.01%) for GFSs and TRIBs for the three glacial floodplains. Boxes show the median (horizontal line), interquartile range (box height), 1.5 x beyond the interquartile range (whiskers), and outliers.

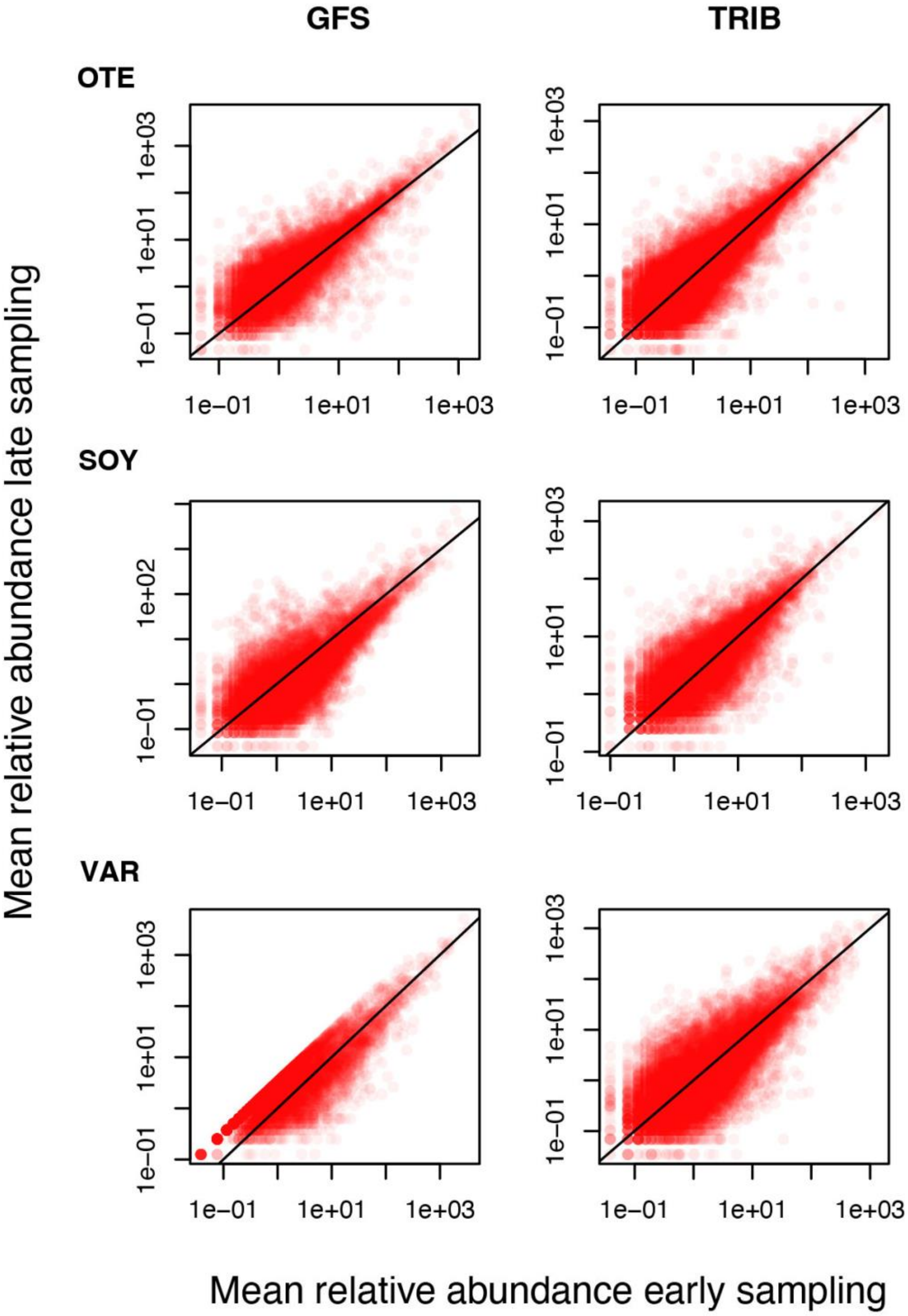

Figure S5. Shown are mean relative abundance of ASVs across all GFS and TRIB samples in OTE, SOY and VAR during the first and second sampling. The black lines represent 1:1 relationship. The figure illustrates the remarkable conservation of rarity and abundance across temporal scales.

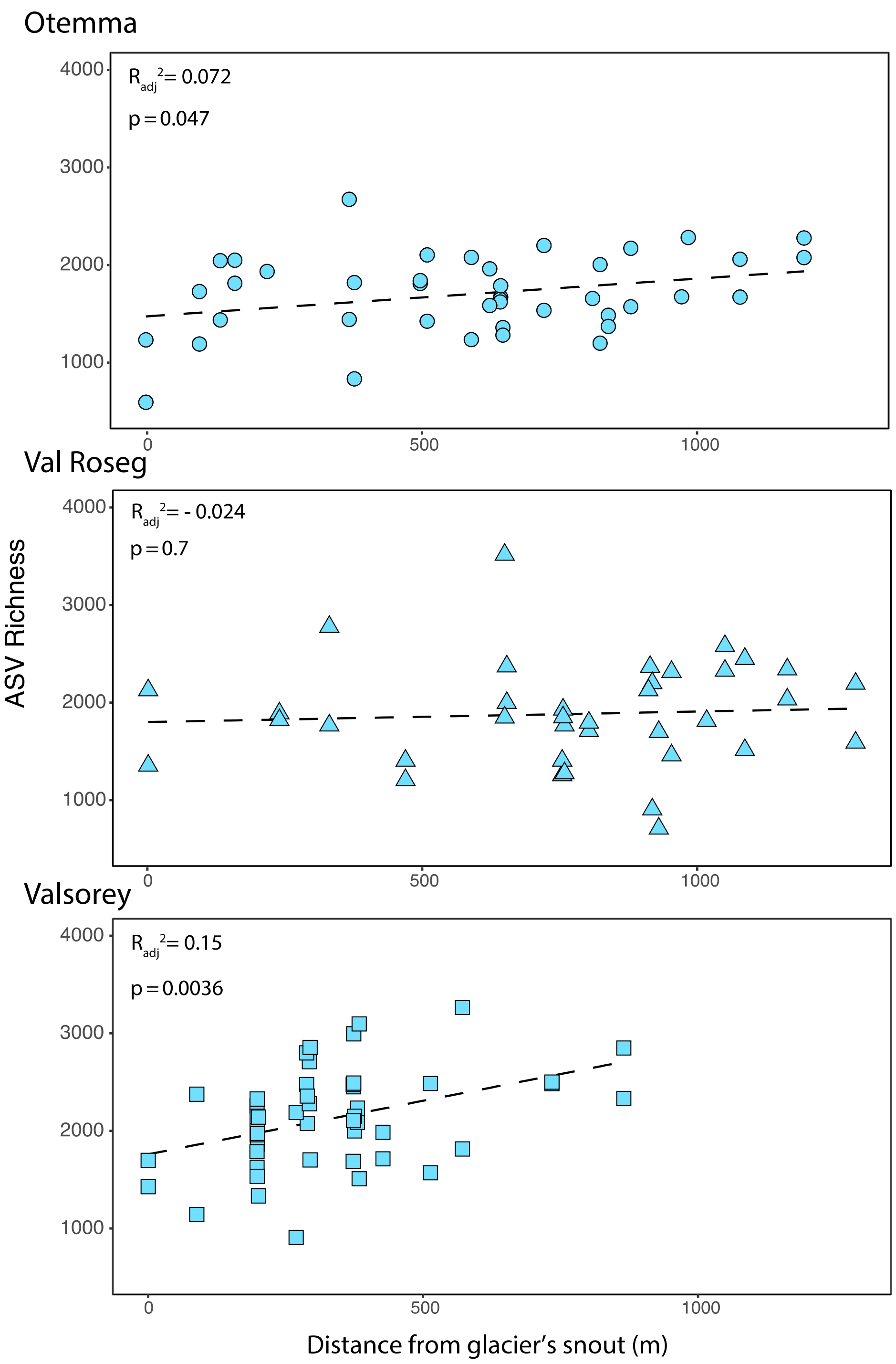

Figure S6. Scatterplots with a linear regression line superimposed showing the number of 16S rRNA AVSs along the longitudinal gradient for glacier-fed samples for the individual floodplains.

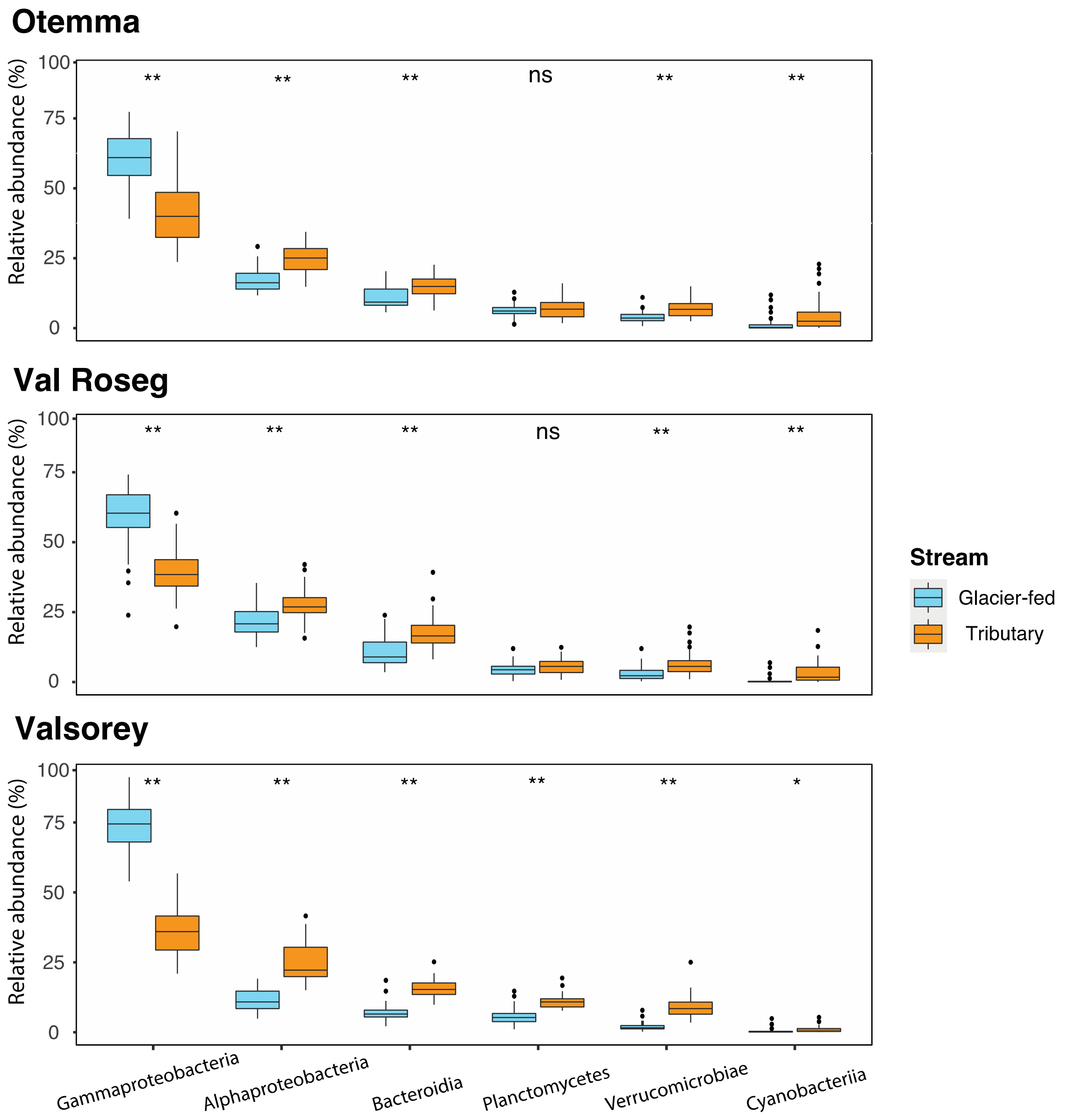

Figure S7. Boxplots showing differences between GFS and TRIBS at the class level for the three glacial floodplains. Significant differences between stream types were tested using t-tests (** $p<0.01$, * $p<0.05$). Boxes show the median (horizontal line), interquartile range (box height), 1.5 x beyond the interquartile range (whiskers), and outliers.

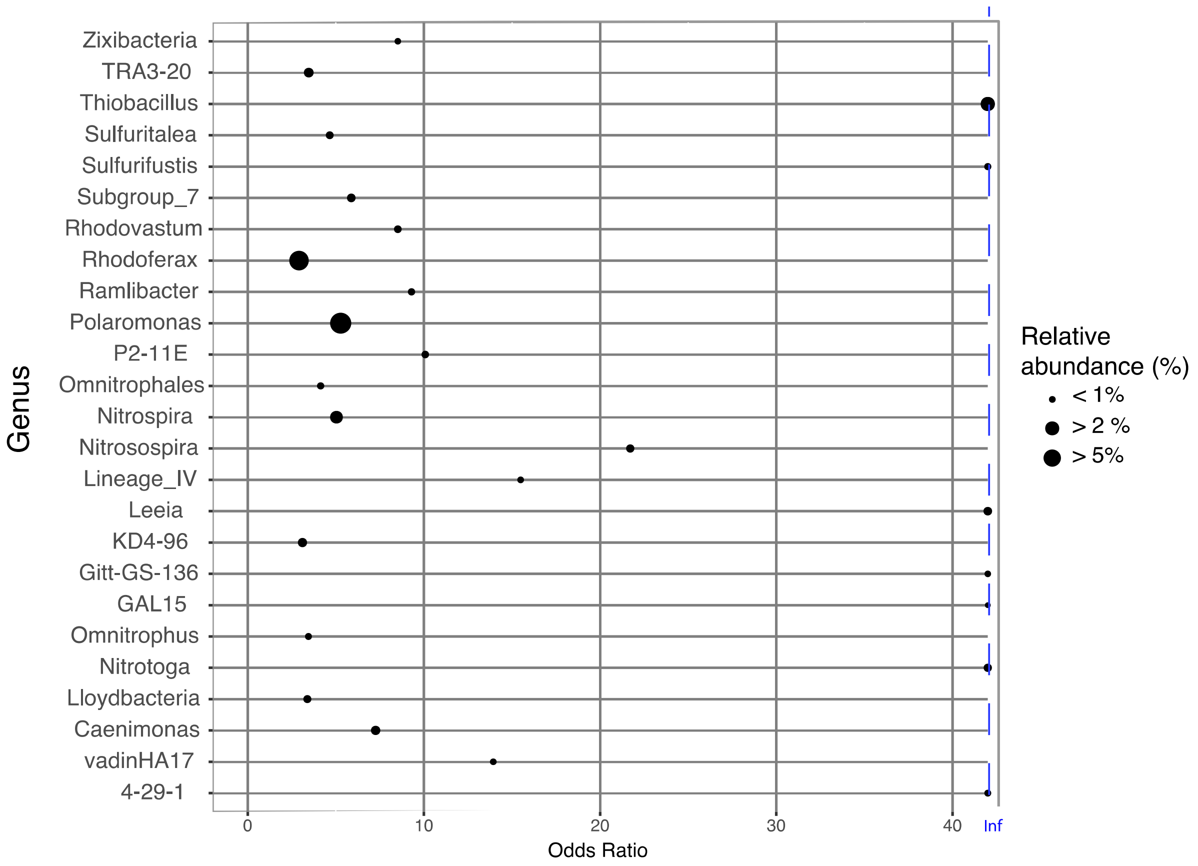

Figure S8. Odd ratio tests identified genera likely to be found in GFS and absent in TRIBs. A total of 25 genera with a significant ($P_{adj}<0.05$) and an Odds ratio >1 are shown here. The blue line represents infinite values.

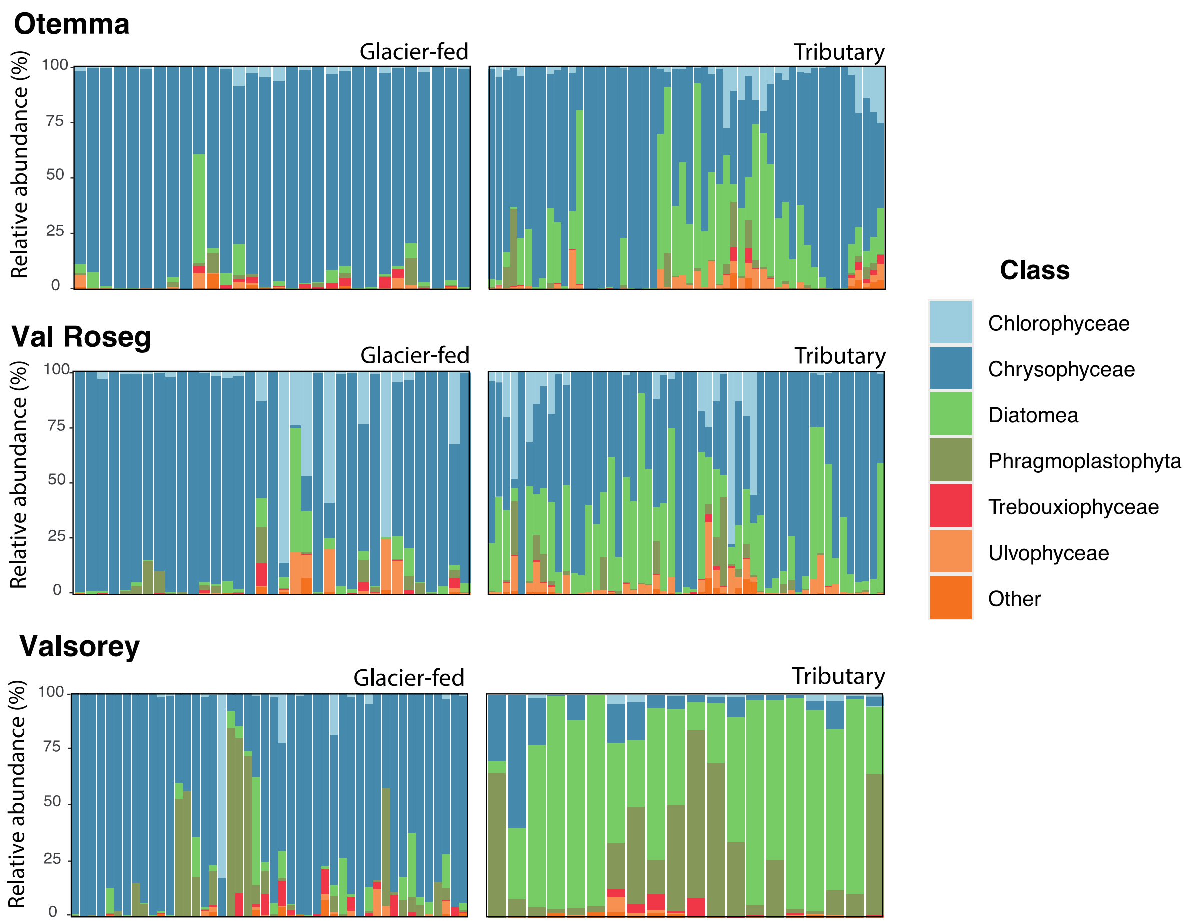

Figure S9. Barplots displaying the taxonomic composition of 18S rRNA gene amplification for phototrophs at the class level for each sample.

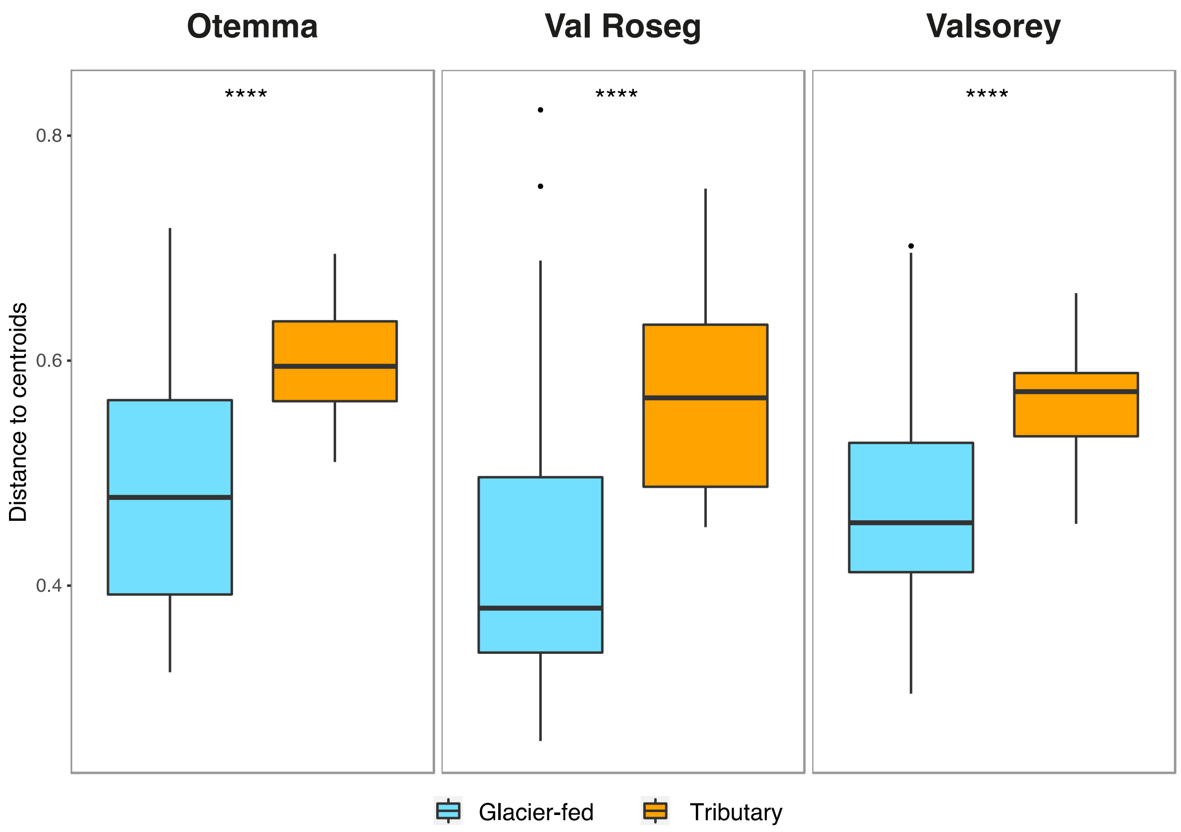

Figure S10. Boxplots based on tests of homogeneity of dispersion analysis representing mean distances from group centroids for 16S community data between stream types. Boxplots' horizontal lines denote median values, boxes represent interquartile ranges (25-75% percentiles), range bars show maximum and minimum extreme values, and hollow circles represent outliers. The significance level is indicated by stars and $p<0.001$.

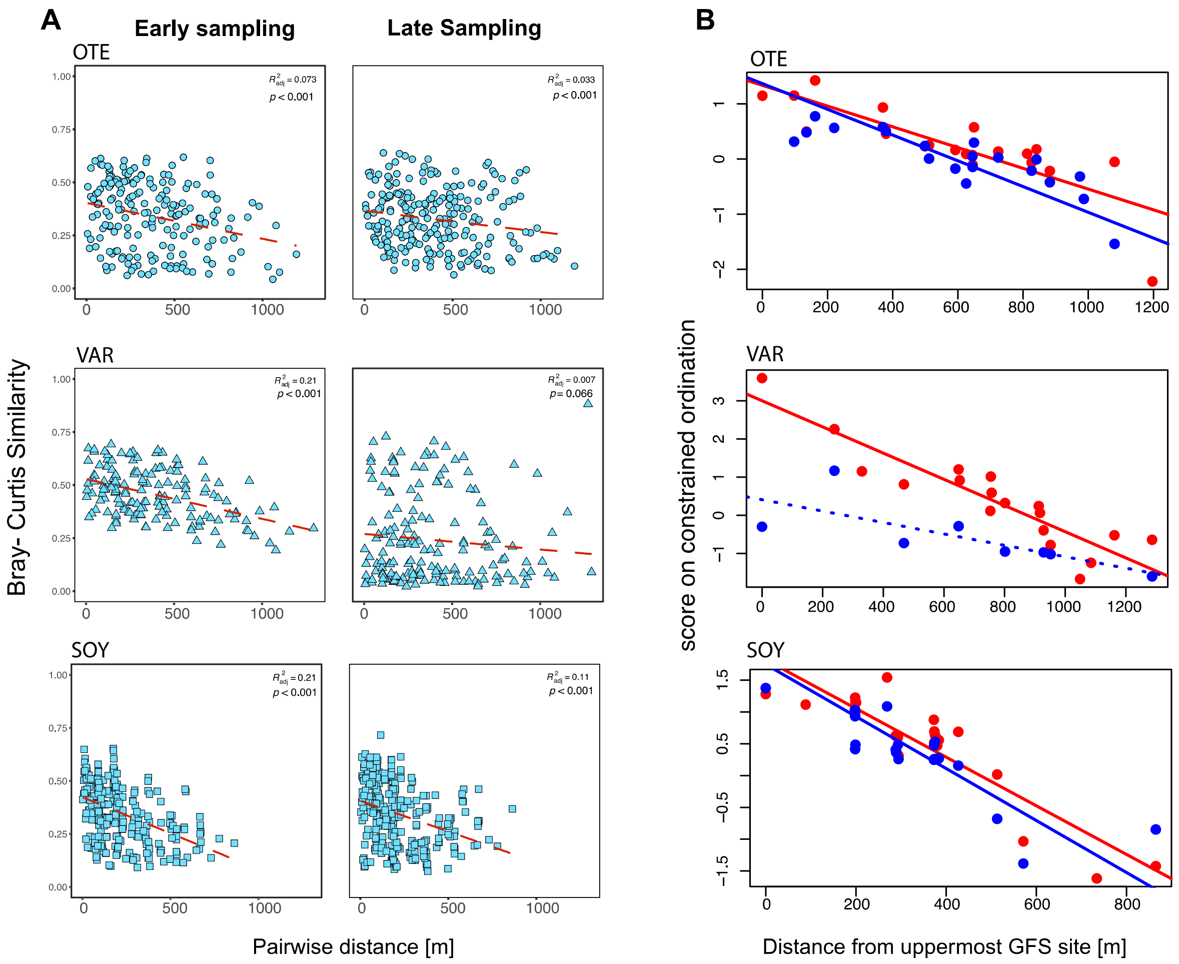

Figure S11. (A) Scatterplots with a linear regression line showing community similarity (Bray-Curtis similarity) against pairwise Euclidean distance between sites. (B) shows site scores for an ordination (CCA) constrained for distance from the glacier snout as a function of distance from the uppermost GFS site. Red symbols represent samples from the first sampling occasion, blue symbols represent samples taken at the second sampling occasion. The regression lines highlight the gradual change in constrained GFS community composition (solid lines for significant linear model fits, p<0.01, dashed line is non-significant).

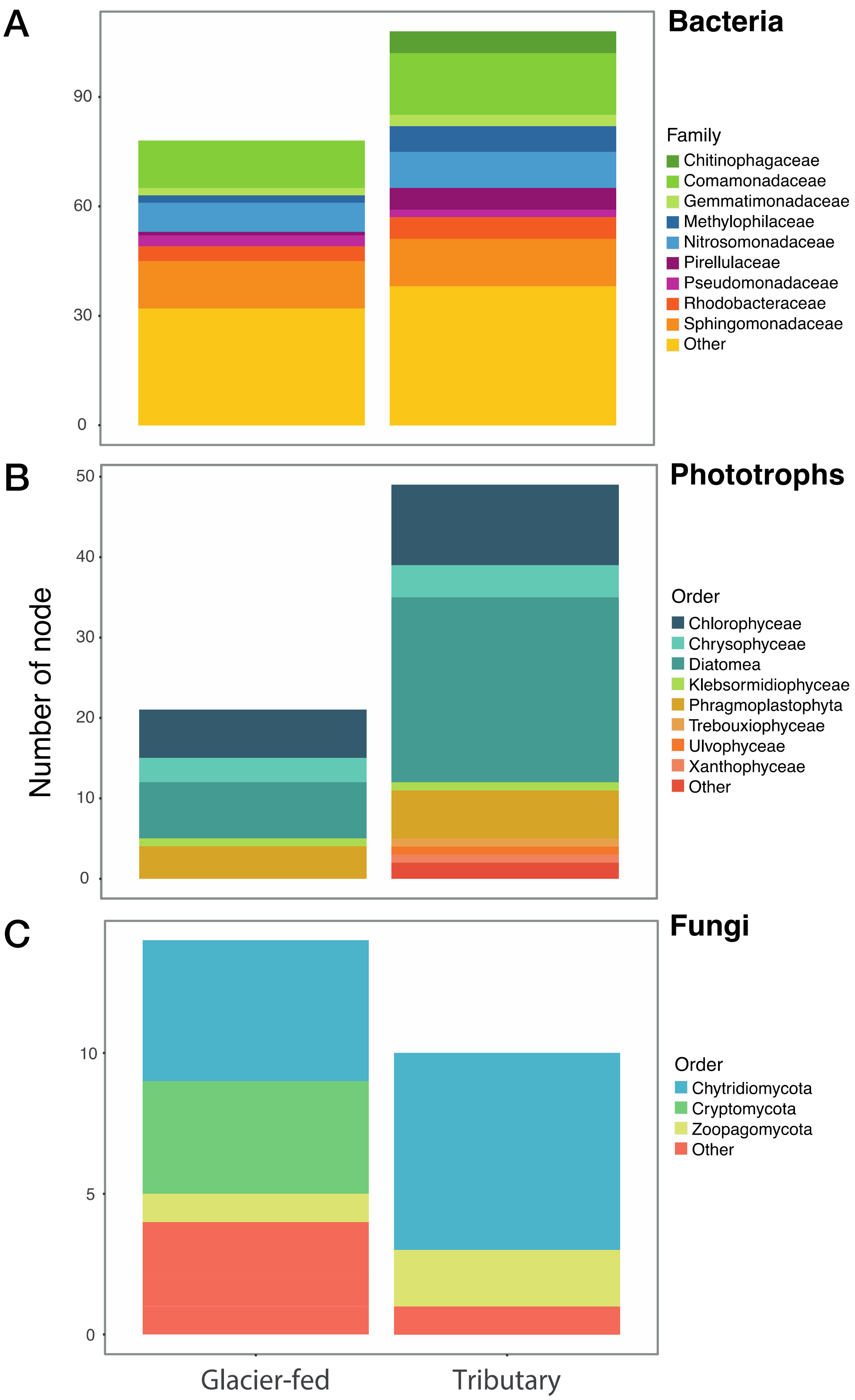

Figure S12. Co-occurrence Network taxonomy bar plot for (A) bacteria, (B) phototrophs, and (C) fungi after all filtering and cleaning stages of network analysis.

2. Supplementary Tables

Table S1. Water samples' physio-chemical characteristics summarised as (mean ± standard deviation) for all three glacial floodplains and for glacier-fed and tributary streams.

		**n**	**pH**	**Cond.**	**Temp.**	**Turb.**	**DOC**	**PO_4-P**	**NH_3-N**	**NO_x-N**	**Alka.**
			-	uS/cm	°C	NTU	ppb	ppb	ppb	ppb	mg/L
**Otemma**	GFS	12	7.59± 0.52	22.3± 9.57	4.94± 4.20	1051± 1003	198± 71.6	5.83± 2.90	22.4± 7.19	118± 48.8	5.61± 2.78
	TRIB	15	7.86± 0.49	48.1± 27.7	12.2± 4.66	30.3± 28.8	379± 241	1.43± 1.27	12.7± 5.98	80.2± 48.2	14.8± 5.49
**Val Roseg**	GFS	6	7.97± 0.43	57.2± 28.4	4.02± 3.08	404± 358	200± 21.8	4.31± 3.21	30.4± 14.9	179± 45.8	16.3± 4.90
	TRIB	21	8.38± 0.448	69.2± 337.2	9.79± 3.68	56.2± 63.8	430± 217	1.85± 1.58	13.3± 3.19	81.4± 68.2	27.1± 16.3
**Valsorey**	GFS	10	8.32± 0.21	190± 58.3	4.38± 2.32	1176± 565	140± 19.4	3.97± 8.32	38.0± 18.5	112± 49.3	50± 17.9
	TRIB	14	8.31± 0.13	248± 69.6	4.45± 2.91	149± 289	158± 59.6	1.10± 0.43	10.6± 3.33	87.6± 33.4	79.9± 17.7

Table S2. ANOVA result table on a selection of water parameters. Testing the effect of Season (Early and Late time point), Glacier (Otemma, Val Roseg, Valsorey), and Stream (Glacier-fed and Tributary), and df: degrees of freedom; SS: Sum of squares; MS: mean sum of squares; Pseudo-*F*: F value by permutation; Pr(>F): p-values. In bold are significant p-values (p<0.05).

Parameter tested	Source of variation	df	SS	MS	Pseudo-*F*	Pr(>F)
Temperature						
	Season	1	11.8	11.8	0.752	0.388645
	Glacier	2	309.7	154.9	9.896	**$1.57*10^{-4}$**
	Stream	1	338.2	338.2	21.612	**$1.45*10^{-5}$**
	Residuals	73	$1.14*10^{3}$	15.6		
Turbidity						
	Season	1	$4.06*10^{4}$	$4.06*10^{4}$	0.172	0.67951
	Glacier	2	$2.93*10^{6}$	$1.47*10^{6}$	6.207	**$3.24*10^{-3}$**
	Stream	1	$1.21*10^{7}$	$1.21*10^{7}$	51.107	**$5.51*10^{-10}$**
	Residuals	73	$1.72*10^{7}$	$2.36*10^{5}$		
Conductivity						
	Season	1	$2.99*10^{3}$	$2.99*10^{3}$	1.612	0.20826
	Glacier	2	$5.1*10^{5}$	$2.55*10^{5}$	137.808	**<2e-16**
	Stream	1	$1.93*10^{4}$	$1.93*10^{4}$	10.408	**$1.9*10^{-3}$**
	Residuals	73	$1.35*10^{5}$	$1.853*10^{3}$		
Dissolved Organic Carbon (DOC)						
	Season	1	$4.04*10^{4}$	$4.04*10^{4}$	1.493	0.226
	Glacier	2	$6.56*10^{5}$	$3.28 *10^{5}$	12.137	**$2.81*10^{-5}$**
	Stream	1	$3.33*10^{5}$	$3.33*10^{5}$	12.334	**$7.67*10^{-4}$**
	Residuals	73	$1.97*10^{6}$	$2.7*10^{4}$		
Dissoved Inorganic Nitrogen (DIN)						
	Season	1	34	34	0.011	0.918
	Glacier	2	661	330	0.103	0.902
	Stream	1	$7.75*10^{4}$	$7.75*10^{4}$	24.150	**$5.3*10^{-6}$**
	Residuals	73	$2.34*10^{5}$	$3.21*10^{3}$		
Soluble Reactive Phosphorous (SRP)						
	Season	1	36.7	36.70	3.284	0.0741
	Glacier	2	18.2	59.12	0.816	0.4460
	Stream	1	195.5	195.54	17.497	**$7.91*10^{-5}$**
	Residuals	73	815.8	11.18		

Table S3. Selection of water physio-chemical indicators for the most upstream and downstream sites of glacier-fed stream for each glacier floodplain (mean ±standard deviation). A Welch Two Sample t-test was used to test the significant difference between Up and Down samples and significant P-values are reported in bold.

	Otemma			Val Roseg			Valsorey		
	Up	Down	P-value	Up	Down	P-value	Up	Down	P-value
Temperature (°C)	0.8 ±0.42	5.47 ±0.64	**0.002**	1.65 ±0.5	5.2 ±3.18	0.1096	3.1 ±1.84	2.4±0.42	0.1568
Turbidity (NTU)	2254 ±557	1343 ±533	0.1989	427 ±482	393 ±369	0.9392	975 ±283	888 ±696	0.8906
Conductivity (uS/cm)	17.8 ±0.5	31.6 ±1.92	**0.003**	55.6 ±35.1	58.1 ±30.5	0.9396	196 ±40	254 ±7.07	0.28
Dissolved Organic Carbon (ppb)	175 ±3.3	219 ±48.3	0.2496	189 ±36.9	206 ±14.6	0.6333	142 ±11.9	154 ±38.9	0.7526
Soluble Reactive Phosphorous (ppb)	7.59 ±1.94	7.69 ±2.6	0.9645	4.98 ±5.43	3.97 ±2.62	0.839	0.99 ±0.13	1.6 ±0.43	0.2699
Dissolved Inorganic Nitrogen (ppb)	117 ±75.8	166 ±26.6	0.5244	182 ±21.3	224 ±44.4	0.1954	139 ±88.4	140 ±43.9	0.9882

Welch Two sample t-test, two-sided. Significant difference between Up and Down sites is indicated by P-value and P-value <0.05 are in bold.

Table S4. ANOVA result table on biomass indicators. Testing the effect of Season (Early and Late time point), Stream (Glacier-fed and Tributary), and Glacier (Otemma, Val Roseg, Valsorey). df: degrees of freedom; SS: Sum of squares; MS: mean sum of squares; Pseudo-*F*: F value by permutation; Pr(>F): p-values. In bold are significant P-values. Chl-a: Chlorophyll-a; BA: Bacterial Abundance; BCP: Bacterial Carbon Production; EPS: Extracellular Polymeric Substance

**Parameter tested**	**Source of variation**	**df**	**SS**	**MS**	**Pseudo-*F***	**Pr(>F)**
Chlorophyll-a						
	Season	1	1.87	1.867	3.487	0.063
	Glacier	2	1.36	0.682	1.273	0.282
	Stream	1	18.32	18.320	34.217	$\mathbf{1.58*10^{-8}}$
	Residuals	243	130.10	0.535		
Bacterial Abundance						
	Season	1	$5.14*10^{14}$	$5.14*10^{14}$	0.344	0.558
	Glacier	2	$6.24*10^{15}$	$3.12*10^{15}$	2.089	0.126
	Stream	1	$9.79*10^{16}$	$9.79*10^{16}$	65.487	$\mathbf{2.8*10^{-14}}$
	Residuals	243	$3.63*10^{17}$	$1.49*10^{15}$		
Bacterial Carbon Production						
	Season	1	0	$2.0*10^{-8}$	0.003	0.957
	Glacier	2	$1.6*10^{-4}$	$8.18*10^{-5}$	15.109	$\mathbf{6.53*10^{-7}}$
	Stream	1	$1.43*10^{-4}$	$1.43*10^{-4}$	26.511	$\mathbf{5.41*10^{-7}}$

	Residuals	243	$1.31*10^{-3}$	$5.41*10^{-6}$		
Extracellular Polymerase Substances						
	Season	1	23	23	0.189	0.664
	Glacier	2	$1.13*10^{3}$	$5.65*10^{3}$	45.736	**$<2*10^{-16}$**
	Stream	1	448	$4.48*10^{2}$	3.624	0.0581
	Residuals	243	$30.0*10^{3}$	$1.24*10^{2}$		

Table S5. perMANOVA results based on Bray-Curtis dissimilarities using 16S abundance data for community structure. Testing the effect of Season (Early and Late time point), Stream (Glacier-fed and Tributary), and Glacier (Otemma, Val Roseg, Valsorey). df: degrees of freedom; SS: Sum of squares; MS: mean sum of squares; Pseudo-*F*: F value by permutation; R^2: portion of variance explained; P(perm): p-values based on 999 permutations.

Source of variation	df	SS	MS	Pseudo-*F*	R^2	P(perm)
Glacier	2	9.172	4.586	14.639	0.093	0.001
Stream type	1	9.572	9.572	30.555	0.097	0.001
Season	1	0.639	0.639	2.040	0.006	0.009
Chlorophyll-*a*	1	2.037	2.0368	6.6477	0.021	0.001
Residuals	251	76.906	0.306		0.782	
Total	256	98.326			1.000	
